# Slowing fetal growth velocity from the mid-trimester may signal increased risks of perinatal morbidity and mortality: a retrospective cohort study

**DOI:** 10.1186/s12916-025-04117-8

**Published:** 2025-05-28

**Authors:** Chloe Jamieson-Grigg, Pawel Kalinowski, Stephen Tong, Esther Turner, Sarah A. Banting, Susan P. Walker, Teresa M. MacDonald

**Affiliations:** 1 Department of Obstetrics, Gynaecology and Newborn Health, University of Melbourne, Melbourne, VIC Australia; 2 Translational Neurodegeneration Group, The Florey Institute of Neuroscience and Mental Health, University of Melbourne, Melbourne, VIC Australia; 3 Mercy Perinatal, Mercy Hospital for Women, Melbourne, VIC Australia

**Keywords:** Adverse perinatal outcome, Appropriate-for-gestational-age, Fetal growth velocity, Fetal growth restriction, Perinatal mortality, Uteroplacental insufficiency, Slowing fetal growth, Small-for-gestational-age, Stillbirth

## Abstract

**Background:**

Undetected fetal growth restriction is a major risk factor for stillbirth. Detecting small babies is a cornerstone of obstetric care, but we fail to detect most uteroplacental insufficiency impairing fetal growth, and most small fetuses. Slowing fetal growth is thought to flag fetal growth restriction, but uncertainty about what constitutes poor growth has hindered clinical translation. We aim to validate slowing fetal growth velocity as a measurable risk factor for adverse pregnancy outcomes, and to better define growth velocity assessment to aid clinical interpretation.

**Methods:**

We performed a retrospective cohort study of ultrasound and birth outcome data. All patients with singleton pregnancies and at least two ultrasound fetal size assessments between 18^+0^ and 39^+6^ weeks, from January 2009 to May 2022, were included. Universal third trimester ultrasound is not performed at our institution; hence, all pregnancies were referred for at least one scan. Primary outcomes were perinatal mortality (stillbirth or neonatal death) and a composite of adverse perinatal outcomes. Fetal growth velocity was calculated between first and last scans, standardized as exact estimated fetal weight (EFW) *z*-score change per week.

**Results:**

Among 24,395 pregnancies, most first scans were routine mid-trimester ultrasounds (median 20^+4^ weeks), with a median 12^+3^ weeks between first and last scans. Each *z*-score/week reduction in EFW growth rate increased perinatal mortality 23-fold (odds ratio (OR) (95% confidence interval (CI)) = 23.25 (7.03–66.45), *p* < 10^−7^), and adverse perinatal outcome 17-fold (OR (95% CI) = 17.54 (12.93–23.84), *p* < 10^−74^). Slowing fetal growth as EFW z-score change/week was associated with adverse perinatal outcome even among those with fetal size considered normal (Hadlock EFW ≥ 10th centile) at last scan, and when confined to term births (OR (95% CI) = 2.35 (1.66–3.33), *p* < 10^−5^; OR (95% CI) = 3.17 (2.10–4.76), *p* < 10^−7^, respectively). A growth rate cut-off of − 0.13 EFW *z*-scores/week was identified as optimal for perinatal mortality by Youden Index. Growth slower than this was associated with sixfold increased odds of perinatal death (OR (95% CI) = 6.40 (3.91–10.30), *p* < 10^−18^).

**Conclusions:**

Slowing fetal growth velocity identifies pregnancies at increased risk of poor outcomes. A slowing growth rate < − 0.13 *z*-scores/week may represent a pragmatic clinical threshold. Fetal growth rate between scans could be incorporated into ultrasound reporting to better identify fetuses at risk.

**Supplementary Information:**

The online version contains supplementary material available at 10.1186/s12916-025-04117-8.

## Background

Stillbirth is a global human tragedy, claiming 2 million lives each year [1]. Most stillbirths, and many neonatal deaths, are due to placental dysfunction [2, 3]. In most cases of fetal growth restriction, there is undersupply to the placenta, or the placenta fails to deliver the nutrients and oxygen required for healthy fetal growth [4]. Collectively, these are referred to as “uteroplacental insufficiency.” Fetal conditions contribute to a minority of cases [4]. Fetal growth restriction (FGR; defined as a fetus that fails to reach its biological growth potential [5]) is a major cause of perinatal morbidity and mortality [6–8]. Being small-for-gestational-age (SGA; weight < 10th centile) [6], particularly severely SGA (< 3rd centile), is known to be associated with stillbirth and adverse neonatal outcomes [2, 9, 10]. Significantly, undetected FGR in the antenatal period further doubles stillbirth risk [8, 9]. If FGR is detected, the fetus is known to be at risk: close monitoring [11], and timely birth reduces death and morbidity [12–15].

The unmet challenge is that we detect less than 30% of at-risk babies [8, 15–17]. This includes the 70% of term stillbirths that occur in fetuses considered appropriate-for-gestational-age (AGA, ≥ 10th centile [18, 19]). We fail to reduce stillbirth because we lack reliable tools to identify uteroplacental insufficiency, unable to support healthy fetal growth.

We have previously investigated fetal growth velocity assessment to better detect presumed uteroplacental insufficiency and FGR. We prospectively showed that slowing fetal growth is associated with antenatal, intrapartum, and postnatal features associated with uteroplacental insufficiency. This was found even among term babies not considered small at birth [20–22], and even when growth velocity was measured from the routine mid-trimester morphology scan [21]. However, our prospective cohort was too small to assess for the most important outcome—perinatal death. Here, we interrogate a large retrospective cohort to confirm the relationships between slowing fetal growth and perinatal morbidity and mortality. We aim to precisely identify features of fetal growth velocity assessment associated with these poor outcomes. Identifying the optimal fetal growth parameter, interval between scans, and threshold of slowing growth that portends fetal risk could assist clinical uptake of fetal growth velocity assessment. Improved detection of uteroplacental insufficiency could improve pregnancy care and reduce the risk of perinatal death.

## Methods

### Study population and design

This was a retrospective longitudinal cohort study of ultrasound and birth outcome data from an Australian tertiary maternity hospital, with approximately 6000 births each year. Participants had an estimated due date from 1 January 2009 to 25 May 2022. The study was approved by Mercy Health’s Human Research Ethics Committee (Project 2020–036).

Using the ultrasound reporting system ViewPoint [23], biparietal diameter, abdominal circumference (AC), head circumference, and femur length data were extracted from all singleton pregnancies with ≥ 2 ultrasounds between 18^+0^ and 39^+6^ weeks’ gestation. We calculated fetal growth velocities between the first and last scans for each pregnancy. These were merged with the hospital’s Birthing Outcomes System (BOS) [24] data, including maternal demographics, obstetric data, neonatal demographics, and birth outcomes. While a morphology scan in the mid-trimester is routinely performed in Australia, subsequent growth scans are not. As such, all scans performed after the routine morphology scan were referred for a clinical indication. These indications include gestational diabetes mellitus, advanced maternal age, in vitro fertilization pregnancy, high maternal body mass index, a past history of a small-for-gestational-age infant, reduced fetal movements, symphysis fundal height measurement smaller or larger than expected, and others.

At our institution, the agreed estimated due date for each pregnancy is assigned according to the last menstrual period, or from the first ultrasound scan in which a fetal heart rate is visualized. Either may be assigned (according to estimated certainty of menstrual dates) if the dates differ by five days or less. If the difference is more than five days, or the menstrual cycle is irregular, then the ultrasound estimated due date is adopted. For estimated due date discrepancies between ViewPoint and BOS of less than nine days, we used the BOS due date, as it was likely used for clinical decision-making. For larger estimated due date discrepancies, manual pregnancy record review was undertaken, ensuring consistency with the agreed due date used in the antenatal clinic.

Pregnancies were excluded if there was: no BOS record (patient birthed elsewhere); less than 2 weeks between first and last scans; only one scan performed at ≥ 18 weeks with a live fetus; or a major congenital anomaly [25].

### Calculating fetal growth velocity

We used the Hadlock four-parameter equation utilizing biparietal diameter, head circumference, abdominal circumference, and femur length recorded at each ultrasound to manually calculate estimated fetal weight (EFW) [26]. Hadlock gestation-specific means and standard deviations were used to calculate the EFW z-scores and birthweight centiles according to the exact gestational day of the scan or birth [26, 27]. Chitty AC *z*-scores were calculated [28].

Fetal growth velocities were calculated between the first and last ultrasound scans as EFW, or AC, *z*-score change per day, multiplied by seven to be reported as *z*-score change per week. These standardized growth rates were calculated as $\frac{\text{Last }z-\text{score}-\text{Baseline }z-\text{score}}{\text{Last exam date}-\text{Baseline exam date}}*7$, ensuring adjustment for the exact number of days between scans. Fetuses with no change in *z*-score between the first and last scans of the dataset had a fetal growth velocity of zero. Cases of slowing fetal growth velocity had a negative number describing their growth rate.

### Adverse perinatal outcomes of interest

Our primary outcomes were perinatal mortality (stillbirth or neonatal mortality) and a pre-defined composite of important adverse perinatal outcomes. These were chosen because of their established associations with placental dysfunction and their impacts on short- and long-term fetal and infant health. The composite adverse perinatal outcome (APO) included any of: perinatal mortality (occurs at the highest rate among severe FGR cases [19, 29, 30]), birthweight < 3rd centile (size parameter agreed to be consistent with FGR even in isolation [31]), nursery admission for > 48 h and/or nursery admission with respiratory support required (indicating significant morbidity; more common in small babies [32]; with negative affect on caregiver-infant relationships [33]), and/or 5-min Apgar score < 7 (indicates poor condition at birth; associated with death and long-term adverse outcomes [34]). Secondary outcomes were SGA (< 10th centile) and severe SGA (< 3rd centile) birthweights.

### Statistical analysis

We analyzed the associations between EFW and AC growth velocities between first and last scan as continuous variables against our binary outcomes of interest using logistic regression. This determined the odds of the outcome per reduction in EFW or AC *z*-score/week growth rate. The odds of outcomes of interest were additionally calculated according to the loss of an EFW *z*-score over a month (4 weeks) and over a trimester (13-week period) to approximate growth rates more often seen clinically. Subgroup analyses were planned in two pre-specified “lowest risk” cohorts: those with fetuses measured as AGA (EFW ≥ 10th centile) at their last growth scan before birth (large-for-gestational-age fetuses were not excluded); and those whose infants were born at term (≥ 37^+0^ weeks). Finally, we performed multi-variate logistic regression in our whole cohort to assess whether the relationships between fetal growth velocity and adverse perinatal outcomes remained significant after adjusting for other known risk factors. We included baseline EFW *z*-score, maternal age, and body mass index at first antenatal visit as covariates.

We performed a receiver operator characteristic curve analysis for our primary outcomes. We identified the optimal threshold of slowing fetal growth for the outcome of perinatal mortality in the whole cohort using the highest Youden Index to define the cut-point. We then calculated the sensitivity, specificity, and positive and negative predictive values for our primary outcomes, and the odds ratio, using chi-square testing, at that threshold. To determine if fetal growth velocity is equally valuable when assessed across different gestational windows, we performed an additional sensitivity analysis. This compared the Youden cut-point’s odds ratio for APO when growth velocity was measured between a first scan performed at 18^+0^–23^+0^ weeks, and a second performed at 25^+0^–30^+0^ weeks; to growth velocity measured between the 25^+0^–30^+0^ week scan and one performed at 32^+0^–37^+0^ weeks. This was performed in a sub-cohort of pregnancies that had three scans performed, with one performed in each of those gestational timeframes.

Finally, we investigated whether fetal growth velocity assessment could be clinically useful even with short intervals between scans. For this, we analyzed fetal growth velocities calculated over specific intervals of two (14–20 days between scans inclusive) and four (28–34 days) weeks—against the primary outcome of perinatal mortality.

Statistical significance was set at *p* < 0.05. Analyses were performed using Microsoft Excel [35] and R (v4.2.0) [36].

## Results

### Cohort characteristics

There were 24,395 pregnancies where two or more ultrasound scans were performed on live fetuses at 18^+0^–39^+6^ weeks’ gestation, at least 2 weeks apart (Additional file 1: Fig. S1). The median (interquartile range (IQR)) gestations of the first and last scans were 20^+4^ (20^+0^–28^+2^) and 35^+6^ (34^+0^–36^+2^) weeks, respectively. Most (62%) baseline scans were performed at a gestation consistent with the routine mid-trimester morphology ultrasound. There was a median interval of 12^+3^ (6^+1^–15^+3^) weeks between first and last scans. There were 81 perinatal deaths (3.3/1000 births) of which 43 were stillbirths (1.8/1000 births). 4140 (17%) pregnancies had an adverse perinatal outcome (Table 1). Compared to pregnancies with surviving infants, pregnancies with perinatal deaths had more frequent, earlier scans; higher rates of preeclampsia; and delivered their smaller babies earlier (Table 1).

**Table 1:** Maternal characteristics, ultrasound, and pregnancy outcomes; with a comparison between pregnancies with perinatal mortalities and those with surviving infants

	**Whole cohort** **(*n* = 24,395)**	**Surviving infants (*n* = 24,314)**	**Perinatal mortality (*n* = 81)**	***p***
**Maternal age (years)**	32.7 (5.1)	32.7 (5.1)	32.5 (5.2)	0.834
**Maternal BMI (kg/m**^**2**^**) at first visit**	25.0 (22.0–30.0)	25.0 (22.0–30.0)	26.0 (23.0–30.0)	0.357
**Parity**	1 (0–1)	1 (0–1)	1 (0–2)	0.352
**Gestational diabetes mellitus**	5280 (21.6%)	5271 (21.7%)	9 (11.1%)	0.030
**Preeclampsia**	924 (3.8%)	914 (3.8%)	10 (12.3%)	< 10^−3^
**Number of scans**	2 (2–3)	2 (2–3)	3 (2–4)	0.015
**First scan gestation (weeks)**	20^+5^ (20^+1^–28^+2^)	20^+5^ (20^+1^–28^+2^)	20^+3^ (19^+5^–22^+3^)	< 10^−4^
**EFW *z*-score at first scan**	0.216 (0.943)	0.218 (0.940)	− 0.260 (1.575)	0.008
**AC *z*-score at first scan**	0.196 (0.905)	0.197 (0.901)	− 0.259 (1.712)	0.019
**Last scan gestation (weeks)**	35^+6^ (34^+0^–36^+2^)	35^+6^ (34^+0^–36^+2^)	28^+3^ (25^+1^–33^+6^)	< 10^−90^
**EFW* z*-score at last scan**	0.107 (1.093)	0.109 (1.087)	− 0.525 (2.088)	0.008
**AC *z*-score at last scan**	0.461 (1.158)	0.463 (1.151)	− 0.146 (2.417)	0.026
**EFW *z*-score change per trimester**	− 0.122 (1.458)	− 0.119 (1.449)	− 0.978 (3.040)	0.013
**AC *z*-score change per trimester**	0.282 (1.733)	0.284 (1.720)	− 0.373 (3.984)	0.141
**Birth gestation (weeks)**	38^+6^ (38^+0^–39^+6^)	38^+6^ (38^+0^–39^+6^)	30^+4^ (26^+6^–37^+0^)	< 10^−238^
**Birthweight (g)**	3240.0 (607.0)	3245.0 (597.7)	1728.8 (1208.3)	< 10^−111^
**Birthweight centile**	36.8 (16.0–64.8)	36.8 (16.0–64.8)	27.6 (3.1–60.3)	0.017
**Composite adverse perinatal outcome**	4140 (17.0%)	4059 (16.7%)	81 (100%)	< 10^−86^
* Perinatal mortality*	*81 (0.3%)*	*0 (0.0%)*	*81 (100%)*	-
* Birthweight* < *3rd centile*	*1214 (5.0%)*	*1194 (4.9%)*	*20 (24.7%)*	-
* Nursery admission* > *48 h*	*2873 (11.8%)*	*2855 (11.7%)*	*18 (22.2%)*	-
* Nursery admission with respiratory support*	*1617 (6.6%)*	*1591 (6.5%)*	*26 (32.1%)*	-
* 5-min Apgar* < *7*	*669 (2.7%)*	*599 (2.5%)*	*70 (86.4%)*	-
**Small-for-gestational-age**	4008 (16.4%)	3977 (16.4%)	31 (38.3%)	< 10^−6^
**Birthweight < 3rd centile**	1214 (5.0%)	1194 (4.9%)	20 (24.7%)	< 10^−14^

Parametric data analyzed by *t*-test and presented as mean (standard deviation). Non-parametric data analyzed by Wilcoxon rank-sum for median (interquartile range). Categorical variables analyzed by chi-square testing, and reported as number (%)

*BMI* Body mass index, *EFW* Estimated fetal weight, *AC* Abdominal circumference, small-for-gestational-age = birthweight < 10th centile

*P*-values are not reported for each individual component of the composite given these were not compared individually

### Fetal growth velocity parameters and adverse perinatal outcome

Slowing fetal growth velocity, measured as ultrasound estimated fetal weight (EFW) *z*-score change per week, was significantly associated with perinatal mortality and morbidity. Each single* z*-score per week reduction in EFW growth rate increased the odds of perinatal mortality 23 times, and the odds of our composite adverse perinatal outcome (APO) more than 17-fold (Table 2). It was consistently and strongly associated with our secondary outcomes—birth of a small infant—and with each component of the pre-specified adverse perinatal outcome composite (Table 2). Slowing fetal abdominal circumference (AC) growth velocity, as measured by ultrasound, was also consistently and strongly associated with perinatal mortality, our composite APO, and birth of a small infant, but not as strongly as slowing EFW growth velocity (Table 2). AC growth velocity had lower odds ratios and slightly higher *p*-values for each outcome of interest. Hence, it appears EFW *z*-score change per week is superior to AC *z*-score change per week in predicting risk. Thus, EFW *z*-score/week was used for all subsequent analyses.

**Table 2:** Odds ratios for adverse perinatal outcomes per unit of slowing fetal growth velocity (using EFW and AC *z*-score change per week, per month (4 weeks), and per trimester (13 weeks)) in the whole cohort

Outcome	Fetal growth velocity parameter	OR (95% CI) of the outcome for each unit reduction in growth velocity	*p*
**Perinatal mortality**	EFW *z*-score/week EFW *z*-score/month EFW *z*-score/trimester	23.25 (7.03–66.45) 2.20 (1.63–2.86) 1.27 (1.16–1.38)	< 10^−7^
	AC *z*-score/week AC *z*-score/month AC *z*-score/trimester	10.40 (2.63–34.21) 1.80 (1.27–2.42) 1.20 (1.08–1.31)	0.0003
**Composite adverse perinatal outcome**	EFW *z*-score/week EFW *z*-score/month EFW *z*-score/trimester	17.54 (12.93–23.84) 2.05 (1.90–2.21) 1.25 (1.22–1.28)	< 10^−74^
	AC *z*-score/week AC *z*-score/month AC *z*-score/trimester	7.10 (5.55–9.10) 1.63 (1.54–1.74) 1.16 (1.14–1.19)	< 10^−53^
* Nursery admission* > *48 h*	EFW *z*-score/week	22.80 (16.20–32.15)	< 10^−70^
	AC *z*-score/week	8.12 (6.16–10.72)	< 10^−48^
* Nursery admission with respiratory support*	EFW *z*-score/week	8.94 (5.92–13.46)	< 10^−24^
	AC *z*-score/week	3.76 (2.62–5.35)	< 10^−12^
* 5-min Apgar* < *7*	EFW *z*-score/week	3.92 (2.08–7.18)	< 10^−4^
	AC *z*-score/week	1.79 (1.01–3.12)	0.0426
**Small-for-gestational-age**	EFW *z*-score/week EFW *z*-score/month EFW *z*-score/trimester	118.30 (84.87–165.43) 3.30 (3.04–3.59) 1.44 (1.41–1.48)	< 10^−172^
	AC *z*-score/week AC *z*-score/month AC *z*-score/trimester	42.70 (32.79–55.77) 2.56 (2.39–2.73) 1.34 (1.31–1.36)	< 10^−168^
**Birthweight < 3rd centile**	EFW *z*-score/week EFW *z*-score/month EFW *z*-score/trimester	254.19 (160.05–405.02) 3.99 (3.56–4.49) 1.53 (1.48–1.59)	< 10^−120^
	AC *z*-score/week AC *z*-score/month AC *z*-score/trimester	88.47 (61.82–126.82) 3.07 (2.80–3.36) 1.41 (1.37–1.45)	< 10^−131^

*OR* Odds ratio, *CI* Confidence interval, small-for-gestational-age = birthweight < 10th centile, *EFW* Estimated fetal weight, *AC* Abdominal circumference

### Adjustment for potential confounders

We performed multi-variate logistic regression adjusting for baseline EFW z-score, maternal age, and maternal body mass index. When these analyses were performed, slowing EFW *z*-score change per week was even more strongly associated with our primary outcomes (Additional file 2: Table S1). The adjusted odds for perinatal mortality and composite adverse perinatal outcome per unit reduction in EFW *z*-score/week growth velocity were as follows: OR (95% confidence interval) = 101.66 (22.13–389.68], *p* < 10^−9^, and OR (95% confidence interval) = 51.49 (36.74–72.36), *p* < 10^−114^, respectively.

### Analyses among appropriate-for-gestational-age at last scan prior to birth, and term birth only, subgroups

We interrogated EFW *z*-score change per week in our pre-planned “low risk” sub-groups. In our cohort, 22,396 (91.8%) pregnancies had a fetus classified as appropriate-for-gestational-age (AGA, EFW ≥ 10th centile) at the time of their last ultrasound prior to birth; and 21,787 (89.3%) had a term birth, occurring at ≥ 37^+0^ weeks’ gestation. These sub-cohorts are generally regarded as “low-risk” as neonatal morbidities that occur at a higher rate among small-for-gestational-age or premature infants—each risk factors for poor outcomes—are excluded. This lower risk status was confirmed by the subgroups’ clinical characteristics, summarized in Additional file 2: Table S2.

Among these two low-risk cohorts, slowing fetal growth velocity was strongly and consistently associated with adverse perinatal outcome and with < 10th and < 3rd centile birthweights (*p* < 10^−5^ for all; Additional file 2: Table S3). In both subgroups, the small numbers of perinatal mortalities meant the associations between slowing EFW growth velocity and perinatal mortality were not statistically significant (Additional file 2: Table S3).

### Optimal fetal growth velocity threshold associated with adverse perinatal outcomes

When receiver operator characteristic curve analysis was performed, fetal growth velocity had only modest predictive performance for both perinatal mortality and for our composite adverse perinatal outcome. The areas under the curve were 0.64 and 0.60, respectively (Additional file 3: Fig. S2a, b). Youden Index testing identified a growth rate of −0.13 *z*-scores per week as the optimal cut-point (Additional file 3: Fig. S2a). At this threshold, slowing fetal growth demonstrated 35.8% sensitivity and 92.0% specificity for perinatal mortality. The diagnostic performance of slowing fetal growth at that level for both primary outcomes is summarized in Table 3. When the cohort was dichotomized at the −0.13 EFW *z*-scores/week growth velocity, and chi-square testing performed, slow fetal growth was associated with increased odds of perinatal mortality and adverse perinatal outcome with OR (95% confidence interval) = 6.40 (3.91–10.30), *p* < 10^−18^ and OR (95% confidence interval) = 3.23 (2.92–3.57), *p* < 10^−130^, respectively (Additional file 2: Table S4).

**Table 3:** Diagnostic performance of EFW growth velocity of −0.13 *z*-scores/week for adverse perinatal outcomes

Outcome	Sensitivity	Specificity	PPV	NPV
Perinatal mortality in whole cohort	35.8%	92.0%	0.23%	98.5%
Composite adverse perinatal outcome in whole cohort	17.5%	93.8%	15.2%	63.3%
Perinatal mortality, when 2 weeks between scans	71.1%	74.9%	0.29%	97.9%
Perinatal mortality, when 4 weeks between scans	53.6%	83.1%	0.20%	98.9%

*EFW* Estimated fetal weight, *PPV* Positive predictive value, *NPV* Negative predictive value

We tested the −0.13 EFW *z*-scores/week threshold amongst a smaller cohort of 4686 pregnancies with three scans performed across two distinct gestational epochs. We found that significantly increased odds for APO were seen when fetal growth velocity was assessed across both time points (*p* < 10^−6^ for both). The odds ratios for APO were very similar between both gestational ranges, with almost complete overlap of the confidence intervals. For fetal growth velocity measured between a scan performed at 18^+0^–23^+0^ weeks inclusive and a second scan performed between 25^+0^ and 30^+0^ weeks, slow fetal growth was associated with an odds ratio (95% confidence interval) of 1.67 (1.36–2.04) for APO. This was compared to an odds ratio of 1.61 (1.34–1.94) for slow growth between the scan performed at 25^+0^–30^+0^ weeks and a second scan performed at 32^+0^–37^+0^ weeks.

### Minimum interval between scans to interpret fetal growth velocity

The results thus far highlight that reporting fetal growth velocity as exact EFW *z*-score change/week enables standardized comparisons with varying intervals between scans. A potential limitation of this approach is how to apply these thresholds where inter-scan intervals are short. To determine if growth velocity could still be useful with short inter-scan intervals, we specifically investigated whether EFW *z*-score change/week could add value, and at what threshold, for two common clinical sub-cohorts: those with scans performed only 2 or 4 weeks apart.

There were 5943 pregnancies with scans performed 2 weeks apart (14–20 days inclusive). Slowing fetal growth still remained significantly associated with perinatal mortality in this cohort. For each *z*-score/week reduction in EFW growth rate, perinatal mortality increased almost 14-fold (OR (95% confidence interval) = 13.77 (4.64–38.72), *p* < 10^−5^) (Additional file 2: Table S5). Receiver operator curve testing demonstrated fair predictive ability with an area under the curve of 0.72 (Additional file 3: Fig. S2c). The optimal Youden to identify perinatal mortality in this cohort was at a threshold growth rate of −0.14 EFW *z*-scores/week or slower. This cut-off achieved 71% sensitivity at 77% specificity (Additional file 3: Fig. S2c), and was very similar to the cut-point identified in the whole cohort, of −0.13 EFW *z*-scores/week. This “optimal cut-point” of −0.13 *z*-scores per week was thus applied universally (Table 3). For those with an inter-scan interval of 2 weeks, the sensitivity and specificity achieved were 71% and 75%, respectively (Table 3).

Among 7757 pregnancies with scans performed four weeks (28–34 days inclusive) apart, slowing fetal growth was again associated with perinatal mortality. For each *z*-score/week reduction in EFW growth rate, perinatal mortality increased a massive 490-fold, but with a very wide confidence interval (OR (95% confidence interval) = 490.94 (48.98–4418.91), *p* < 10^−7^) because of the rarity of the outcome (Additional file 2: Table S5). Receiver operator characteristic curve analysis showed that fetal growth velocity performed with moderate discrimination in this cohort with an area under the curve of 0.75 (Additional file 3: Fig. S2d). Here, the Youden Index identified a threshold growth rate of < −0.07 EFW *z*-scores/week as the optimal cut-point—achieving 71% sensitivity and 71% specificity (Additional file 3: Fig. S2d). When the “universal cut-off” of −0.13 EFW *z*-scores/week was applied to this cohort, 54% sensitivity for 83% specificity for perinatal mortality was achieved. The diagnostic performance of our optimal cut-point in all cohorts is summarized in Table 3. These results highlight that growth velocity assessment can still add value despite short inter-scan intervals, and that the cut-point identified of −0.13 EFW *z*-scores/week is reasonable to apply regardless of inter-scan interval.

## Discussion

### Main findings

In this detailed study of 24,395 pregnancies, we report slowing EFW growth velocity to be significantly associated with perinatal mortality. This applies even when fetal size is first measured at the routine mid-trimester ultrasound, which is performed almost universally. This means fetal growth velocity can be included as a “checkpoint of uteroplacental health” in any pregnancy where even one additional scan has been performed. Slowing fetal growth is also consistently and strongly associated with other important adverse perinatal outcomes, even amongst cohorts not traditionally thought of as “at risk.” This includes fetuses reported as normally grown at their last scan prior to birth, and those born at term. For these cohorts, increased surveillance and timely delivery might represent an appropriate, safe, and acceptable intervention to reduce stillbirth [8, 37]. Measuring fetal growth velocity thus adds valuable risk assessment to all pregnancies, including those with apparently normal fetal size.

We have performed the analyses necessary to inform the clinical use of fetal growth velocity assessment, using a standard that can be applied irrespective of inter-scan interval. We found that EFW *z*-score change per week should be used to measure fetal growth velocity as it outperforms AC growth rate in its associations with perinatal mortality and adverse perinatal outcomes. We report a relevant clinical threshold that most effectively identifies increased perinatal mortality risk. Specifically, −0.13 EFW *z*-scores/week or slower is the threshold which performs with optimal balance of sensitivity and specificity, and it seems to perform similarly well across the second and third trimesters.

Finally, we report that fetal growth velocity can flag increased perinatal mortality risk, even when calculated over short intervals. Application of the −0.13 EFW *z*-score/week growth rate cut-off to these specific cohorts still performs with reasonable predictive ability, maintaining high negative predictive value and an appropriate balance of specificity for sensitivity. In our whole cohort, only 8.1% of pregnancies demonstrated slowing fetal growth of this magnitude. Some fetuses were already small, or otherwise known to be at risk. This means the clinical impact of flagging these additional screen-positive pregnancies as higher risk—warranting increased surveillance—would be relatively modest.

### Interpretation of results and comparison with other studies

We add to the growing body of evidence advocating fetal growth velocity as an independent assessment to predict poor outcomes. We previously showed that slowing fetal growth velocity from the mid-trimester is associated with outcomes associated with placental dysfunction [20–22]. These include intrapartum acidosis and low neonatal body fat. An important difference is that the current study has sufficient power to detect rare outcomes, like perinatal mortality and severe morbidity.

Importantly, we have validated slowing growth velocity as a risk factor for adverse perinatal outcomes specifically among fetuses considered normally grown [20, 21, 38]. Detecting slowing fetal growth velocity flags these pregnancies—currently hiding in plain sight—as “at-risk.” This is important given that approximately 70% of term stillbirths occur in this group [18, 19].

Previous studies support a threshold growth rate, adjusting for time between scans, to identify increased risk. These include a study where slow growth was defined as −3.4 EFW centiles/week over ≥ 8 weeks [38]. Slow growth was associated with double the risks of stillbirth, neonatal death, and neonatal intensive care admission [38]. Further, Pacora et al. concluded that antepartum fetal death is preceded by slowing growth, even in those not classified as SGA [18]. Their median growth velocity among fetal deaths was −4.27 EFW centiles/week. However, Pacora’s scans were performed at earlier gestations (14–32 weeks), reflecting earlier-onset FGR. We have assessed fetal growth as *z*-score change per week rather than centile/week to account for the non-uniform spacing between centile lines. Nevertheless, given that centiles are generated from *z*-scores, there are obvious parallels between these results.

Larsen et al. found that slowing fetal growth is associated with perinatal death [39]. However, they found the magnitude of the fall in centile between scans to be significant rather than a slowing growth rate [39]. Specifically, > 50 centiles loss was predictive of perinatal mortality. In our cohort, the magnitudes of EFW or AC centile falls without adjustment for time between scans were not associated with perinatal mortality (data not shown). The Larsen cohort comprised only term births. This difference potentially biased toward longer intervals and larger centile changes between scans. Our study instead allows for the fact that the outcome of term birth is unknown at the time of growth scan. While the International Society of Ultrasound in Obstetrics and Gynaecology also recommends a fall of > 50 centiles plus additional features to define FGR [31], this lacks guidance on the time course for this fall – and fails to adjust for non-uniform spacing between centile lines. This precludes interpretation of growth velocity according to the inter-scan interval. Moreover, we have previously reported that this degree of EFW fall is uncommon [20], and thus this parameter may lack sensitivity.

Aside from the magnitude of centile fall, other slowing fetal growth velocities previously investigated include AC and EFW rates as mm/week [40] and g/week [41], respectively. While these, too, demonstrate associations with adverse perinatal outcomes, we chose *z*-scores, which allow us to adjust for gestational age and for varying average growth rates at different gestations. That EFW *z*-score/week performs better than AC *z*-score/week aligns with recommendations to use multiple fetal measurements rather than AC alone [11, 42].

### Strengths and limitations

Study strengths include our large dataset and inclusive representation of “all-comers” having two or more scans from 18 weeks. The size of the dataset enabled detection of associations between slowing fetal growth velocity and the rare outcome of perinatal mortality. That associations between slowing fetal growth and adverse perinatal outcome were consistently strong confirms that slowing fetal growth might flag increased risk and might help clinicians to better identify uteroplacental insufficiency. Further, we included specific analyses in fetuses thought to be AGA. This group would usually not be flagged as high risk and is a previously under-investigated cohort.

We distil critical information to allow clinical translation of this work. This includes identification of a key threshold to flag concern, and testing to confirm that growth velocity assessment is valuable even with only short intervals between scans. This and other potential thresholds warrant testing with clinicians and consumers, to identify the acceptable trade-off between optimizing sensitivity and minimizing false positive rates. Our method of calculating *z*-score according to exact day of gestation, and growth rate per exact number of days between scans maximizes accuracy. This is known to be important for EFW centile calculation and interpretation [43].

Limitations of this study include its retrospective nature. Only participants with at least one ultrasound assessment in addition to routine antenatal care were included. These pregnancies often have a risk factor for adverse perinatal outcomes requiring additional surveillance, as highlighted by the higher-than-expected rates of small infants in our cohort. This introduces potential bias, which could be reduced if replicated where third-trimester ultrasound is routine. However, despite its retrospective nature, our cohort was largely free of intervention bias since neither mid-trimester EFW *z*-score nor centile, nor fetal growth velocity, are routinely reported in our institution.

Fortunately, perinatal mortality is a rare outcome. However, another limitation is that logistic regression can underestimate the probability of rare events. The relative imbalance between the number of cases and non-cases makes it harder to detect true effects and can create inflated estimates and wide confidence intervals (which we see in the results). To combat this, we checked our results using Firth’s method and found this produced similar odds ratios (data not shown). However, the rarity of perinatal mortality meant that our subgroup analyses were underpowered to detect significant associations between slowing fetal growth velocity and this primary outcome. Despite this, because slowing growth remained significantly and strongly associated with adverse perinatal outcome (a more common event) even in these much lower risk cohorts, we are reassured that it is a valuable measure to calculate at the time of every growth scan. Additionally, due to the exploratory nature of the study, correction for multiple testing was not planned a priori. Validation in larger external cohorts, or through combining standardized datasets, could better quantify risk among these cohorts traditionally considered lower risk.

Finally, there is a risk of over-interpreting sub-analyses of our cohorts with 2 and 4 weeks between scans—as some wide confidence intervals illustrate. These cohorts are smaller, and are likely to be particularly high risk, given more intense growth surveillance with short inter-scan intervals. But, a previous analysis of 102,138 pregnancies has also reported cut-offs in EFW centiles/week to define slow growth for these exact intervals, and has similarly found slow growth to be associated with adverse pregnancy outcomes [38]. In particular, and in support of our findings, they found that slow growth over a short median inter-scan period of 3 weeks and 3 days was predictive of stillbirth, even in fetuses that were not SGA.

### Clinical implications

Fetal growth velocity as an ultrasound indicator of perinatal risk could feasibly be integrated into routine antenatal care. It requires just a single growth scan after the routine mid-trimester morphology scan [21]. Mid-trimester scans are almost universal, and “growth scans” are commonplace. At least 40% of pregnancies undergo a clinically indicated third trimester ultrasound [17]. Growth velocity could be easily and routinely reported at these scans to provide extra information to clinicians regarding perinatal risk. We show that AGA fetuses experiencing slow growth demonstrate outcomes consistent with FGR. They might benefit from surveillance and delivery recommendations traditionally reserved for the SGA [44–46]. Because slowing fetal growth velocity is associated with small birthweights, routine reporting of EFW* z*-score/week growth rate would also likely improve SGA detection [16, 17]. This is known to reduce stillbirth risk [8, 47].

## Conclusions

We have shown that slowing EFW *z*-score/week growth velocity is a likely sign of uteroplacental insufficiency. It is strongly associated with adverse perinatal outcome, including mortality. Calculating fetal growth velocity among fetuses that are not small identifies those hitherto unrecognized as being at risk. Fetal growth velocity assessment can use the routine mid-trimester scan as the baseline fetal size assessment, which means this “checkpoint of uteroplacental health” can be reported where any additional growth scan beyond routine care has been performed. It is thus highly feasible to include fetal growth velocity assessment as a routine part of reporting where ultrasound is performed in the second half of pregnancy. This could improve detection of uteroplacental insufficiency and increased perinatal risk, enabling clinicians to tailor care to reduce that risk.

## Supplementary Information

Additional file 1: Figure S1. Diagram of included and excluded pregnancies in the study. *BOS *Birthing Outcome System; *FDIU *fetal death in utero.

Additional file 2: Table S1. Adjusted odds ratios for adverse perinatal outcomes in the whole cohort after multi-variate analysis. Each individual predictor has been adjusted for the other three co-variates to produce the adjusted odds ratio.*CI *confidence interval, *EFW *estimated fetal weight, *Kg *kilogram, *m *metre. Table S2. Maternal characteristics, scan and pregnancy outcomes in the appropriate-for-gestational age cohort and term cohort. Data presented as mean (standard deviation) or median (interquartile range) depending on distribution for continuous variables and as number (%) for categorical variables. *AGA* appropriate-for-gestational-age, *BMI *body mass index. Small-for-gestational-age = birthweight <10^th^ centile. Table S3. Odds ratios for adverse perinatal outcomes per unit reduction in EFW z-score change per week growth velocity in sub-groups: (i) those measured as AGA at last scan; and (ii) those born at term*AGA *appropriate-for-gestational-age (EFW ³10^th^ centile), *OR *odds ratio, *CI *confidence interval, *EFW *estimated fetal weight. Small-for-gestational-age = birthweight <10^th^ centile. Table S4. Odds ratios for adverse outcomes by Chi Square testing when cohort is dichotomised according to a threshold growth rate of -0.13 EFW z-scores/week. Chi square testing performed. *Slow growth *= EFW growth rate < -0.13 z-scores/week; *Normal growth* = EFW growth rate >-0.13 z-scores/week. Table S5. Odds ratios for perinatal mortality per unit reduction in EFW z-score change per week growth velocity, in the cohorts with short inter-scan intervals*OR *Odds Ratio; *CI *Confidence Interval. “2 weeks” refers to 14-20 days inclusive between scans; “4 weeks” refers to 28-34 days inclusive between scans. 

Additional file 3: Figure S2. ROC curves of slowing EFW z-score/week growth velocity for: a. Perinatal Mortality in the whole cohort; b. Composite adverse perinatal outcome in the whole cohort; c. Perinatal mortality in the cohort with a 2-week inter-scan interval; d. Perinatal mortality in the cohort with a 4-week inter-scan interval. *AUC *Area under curve; *ROC *Receiver Operator Characteristic.

Additional file 4: Completed STROBE Checklist.

## Data Availability

The datasets used and/or analysed in the current study are available from the corresponding author on reasonable request.

## Abbreviations

AC: Abdominal circumference
AGA: Appropriate-for-gestational age
APO: Adverse perinatal outcome
BOS: Birthing Outcome System
BMI: Body mass index
CI: Confidence interval
EFW: Estimated fetal weight
FGR: Fetal growth restriction
IQR: Interquartile range
NPV: Negative predictive value
OR: Odds ratio
PPV: Positive predictive value
SGA: Small-for-gestational age
