# Supplementary figures and images for: Slowing fetal growth velocity from the mid-trimester may signal increased risks of perinatal morbidity and mortality: a retrospective cohort study
Source: BMC Med. 2025 May 28;23:298. doi: 10.1186/s12916-025-04117-8 (PMC12121231; doi:10.1186/s12916-025-04117-8)

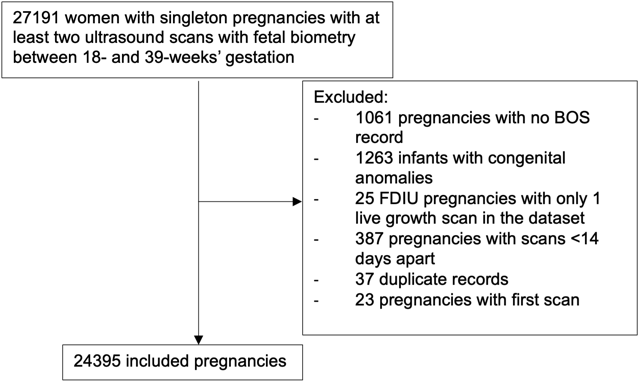

Supplement: Supplementary file 1 — Additional file 1: Figure S1. Diagram of included and excluded pregnancies in the study. BOS Birthing Outcome System; FDIU fetal death in utero. [file 12916_2025_4117_MOESM1_ESM.png]

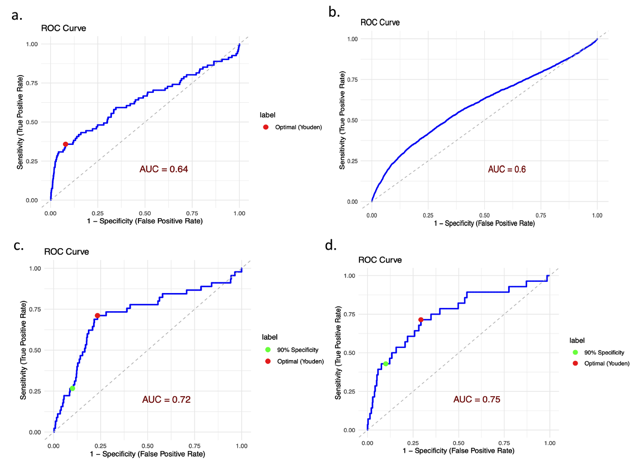

Supplement: Supplementary file 3 — Additional file 3: Figure S2. ROC curves of slowing EFW z-score/week growth velocity for: a. Perinatal Mortality in the whole cohort; b. Composite adverse perinatal outcome in the whole cohort; c. Perinatal mortality in the cohort with a 2-week inter-scan interval; d. Perinatal mortality in the cohort with a 4-week inter-scan interval. AUC Area under curve; ROC Receiver Operator Characteristic. [file 12916_2025_4117_MOESM3_ESM.png]
