# Supplementary material for: Slowing fetal growth velocity from the mid-trimester may signal increased risks of perinatal morbidity and mortality: a retrospective cohort study
Source: BMC Med. 2025 May 28;23:298. doi: 10.1186/s12916-025-04117-8 (PMC12121231; doi:10.1186/s12916-025-04117-8)
Supplement: Supplementary file 2 — Additional file 2: Table S1. Adjusted odds ratios for adverse perinatal outcomes in the whole cohort after multi-variate analysis. Each individual predictor has been adjusted for the other three co-variates to produce the adjusted odds ratio.CI confidence interval, EFW estimated fetal weight, Kg kilogram, m metre. Table S2. Maternal characteristics, scan and pregnancy outcomes in the appropriate-for-gestational age cohort and term cohort. Data presented as mean (standard deviation) or median (interquartile range) depending on distribution for continuous variables and as number (%) for categorical variables. AGA appropriate-for-gestational-age, BMI body mass index. Small-for-gestational-age = birthweight <10th centile. Table S3. Odds ratios for adverse perinatal outcomes per unit reduction in EFW z-score change per week growth velocity in sub-groups: (i) those measured as AGA at last scan; and (ii) those born at termAGA appropriate-for-gestational-age (EFW ³10th centile), OR odds ratio, CI confidence interval, EFW estimated fetal weight. Small-for-gestational-age = birthweight <10th centile. Table S4. Odds ratios for adverse outcomes by Chi Square testing when cohort is dichotomised according to a threshold growth rate of -0.13 EFW z-scores/week. Chi square testing performed. Slow growth = EFW growth rate < -0.13 z-scores/week; Normal growth = EFW growth rate >-0.13 z-scores/week. Table S5. Odds ratios for perinatal mortality per unit reduction in EFW z-score change per week growth velocity, in the cohorts with short inter-scan intervalsOR Odds Ratio; CI Confidence Interval. “2 weeks” refers to 14-20 days inclusive between scans; “4 weeks” refers to 28-34 days inclusive between scans. [file 12916_2025_4117_MOESM2_ESM.docx]

**Table S1.** Adjusted odds ratios for adverse perinatal outcomes in the whole cohort after multi-variate analysis.

| **Outcome** | **Per each:** | **Adjusted odds ratio (95% CI)** | ***p*** |
| --- | --- | --- | --- |
| **Perinatal Mortality** | Unit reduction in EFW z-score/week growth velocity | 101.66 (22.13 – 389.68) | <10^-9^ |
|  | Unit reduction in baseline EFW z-score | 2.01 (1.62 – 2.48) | <10^-9^ |
|  | Year increase in maternal age | 1.02 (0.97 – 1.06) | 0.47 |
|  | Kg/m^2^ increase in maternal body mass index | 1.03 (1.00 – 1.06) | 0.04 |
| **Composite adverse perinatal outcome** | Unit reduction in EFW z-score/week growth velocity | 51.49 (36.74 – 72.36) | <10^-114^ |
|  | Unit reduction in baseline EFW z-score | 1.70 (1.63 – 1.77) | <10^-129^ |
|  | Year increase in maternal age | 0.996 (0.990 – 1.003) | 0.32 |
|  | Kg/m^2^ increase in maternal body mass index | 1.016 (1.010 – 1.021) | <10^-8^ |

Each individual predictor has been adjusted for the other three co-variates to produce the adjusted odds ratio.

*CI* confidence interval, *EFW* estimated fetal weight, *Kg* kilogram, *m* metre

**Table S2.** Maternal characteristics, scan and pregnancy outcomes in the appropriate-for-gestational age cohort and term cohort.

|  | Whole  (n = 24,395) | AGA at last scan  (n = 22,396) | Born at term  (n = 21,787) |
| --- | --- | --- | --- |
| Age (years) | 32.7 (5.1) | 32.7 (5.1) | 32.7 (5.1) |
| Maternal BMI (kg/m^2^) at first visit | 25.0 [22.0 - 30.0] | 25.0 [ 22.0 – 30.0] | 25.0 [22.0 – 30.0] |
| Parity | 1 [0 – 1] | 1 [ 0 – 1] | 1 [ 0 – 1] |
| Gestational diabetes mellitus | 5280 (21.6%) | 4884 (21.8%) | 4835 (22.2%) |
| Preeclampsia | 924 (3.8%) | 745 (3.3%) | 558 (2.6%) |
| Number of scans | 2 [2 – 3] | 2 [ 2 – 3] | 2 [2 – 3] |
| First scan gestation (weeks) | 20^+4^ (20^+0^ – 28^+2^) | 20^+5^ (20^+0^ – 28^+2^) | 20^+5^ (20^+1^ – 28^+3^) |
| Last scan gestation (weeks) | 35^+6^ (34^+0^ – 36^+2^) | 35^+5^ (34^+0^ – 36^+2^) | 36^+0^ (34^+1^ – 36^+3^) |
| Birth gestation (weeks) | 38^+6^ (38^+0^ – 39^+6^) | 39^+0^ (38^+1^ – 39^+6^) | 39^+0^ (38^+2^ – 39^+6^) |
| Birthweight (g) | 3240.0 (607.0) | 3321.4 (541.8) | 3349.3 (484.6) |
| Birthweight centile | 36.8 (16.0 – 64.8) | 40.9 (20.0 – 67.4) | 38.2 (17.1 – 65.6) |
| Perinatal mortality | 81 (0.33%) | 58 (0.26%) | 21 (0.10%) |
| Adverse perinatal outcome | 4140 (17.0%) | 3022 (13.5%) | 2225 (10.2%) |
| Small-for-gestational-age | 4008 (16.4%) | 2446 (10.9%) | 3196 (14.7%) |
| < 3^rd^ centile birthweight | 1214 (5.0%) | 389 (1.7%) | 761 (3.5%) |

Data presented as mean (standard deviation) or median (interquartile range) depending on distribution for continuous variables and as number (%) for categorical variables. *AGA* appropriate-for-gestational-age, *BMI* body mass index. Small-for-gestational-age = birthweight <10^th^ centile.

**Table S3.** Odds ratios for adverse perinatal outcomes per unit reduction in EFW z-score change per week growth velocity in sub-groups: (i) those measured as AGA at last scan; and (ii) those born at term

| Outcome | Sub-group | OR [95% CI] of outcome per unit reduction in EFW centile/week growth velocity | *p* |
| --- | --- | --- | --- |
| Perinatal mortality | AGA at last scan | 1.90 [0.18 – 14.37] | 0.58 |
|  | Born at term | 1.92 [0.04 – 37.93] | 0.75 |
| Composite adverse perinatal outcome | AGA at last scan | 2.35 [1.66 – 3.33] | <10^-5^ |
|  | Born at term | 3.17 [2.10 – 4.76] | <10^-7^ |
| Small-for-gestational-age | AGA at last scan | 10.42 [7.15 – 15.21] | <10^-33^ |
|  | Born at term | 60.20 [40.87 – 88.97] | <10^-93^ |
| < 3^rd^ centile | AGA at last scan | 8.44 [3.92 – 17.42] | <10^-7^ |
|  | Born at term | 53.59 [29.25 – 98.72] | <10^-36^ |

*AGA* appropriate-for-gestational-age (EFW ≥10^th^ centile), *OR* odds ratio, *CI* confidence interval, *EFW* estimated fetal weight. Small-for-gestational-age = birthweight <10^th^ centile.

**Table S4.** Odds ratios for adverse outcomes by Chi Square testing when cohort is dichotomised according to a threshold growth rate of -0.13 EFW z-scores/week.

| **Outcome** | **Number (%) when slow growth**  **(N = 1977)** | **Number (%) when normal growth**  **(N = 22,418)** | **Odds Ratio**  **[95% Confidence Interval]** | ***p*-value** |
| --- | --- | --- | --- | --- |
| **Perinatal Mortality** | 29  (1.47%) | 52  (0.23%) | 6.40  [3.91 – 10.30] | <10^-18^ |
| **Composite adverse perinatal outcome** | 726  (36.72%) | 3,414  (15.23%) | 3.23  [2.92 – 3.57] | <10^-130^ |

Chi square testing performed. *Slow growth* = EFW growth rate < -0.13 z-scores/week; *Normal growth* = EFW growth rate > -0.13 z-scores/week.

**Table S5.** Odds ratios for perinatal mortality per unit reduction in EFW z-score change per week growth velocity, in the cohorts with short inter-scan intervals

| Inter-scan interval | Number | OR [95% CI] for perinatal mortality | *p* |
| --- | --- | --- | --- |
| 2 weeks | 5,943 | 13.77 [4.64 – 38.72] | <10^-5^ |
| 4 weeks | 7,757 | 490.94 [48.98 – 4418.91] | <10^-7^ |

*OR* Odds Ratio; *CI* Confidence Interval. “2 weeks” refers to 14-20 days inclusive between scans; “4 weeks” refers to 28-34 days inclusive between scans.
