# Supplementary material for: Slowing fetal growth velocity from the mid-trimester may signal increased risks of perinatal morbidity and mortality: a retrospective cohort study
Source: BMC Med. 2025 May 28;23:298. doi: 10.1186/s12916-025-04117-8 (PMC12121231; doi:10.1186/s12916-025-04117-8)
Supplement: Supplementary file 4 — Additional file 4: Completed STROBE Checklist. [file 12916_2025_4117_MOESM4_ESM.doc]

STROBE Statement—Checklist of items that should be included in reports of ***cohort studies***

|  | Item No | Recommendation | Checklist |
| --- | --- | --- | --- |
| **Title and abstract** | 1 | (*a*) Indicate the study’s design with a commonly used term in the title or the abstract | Title, page 1 |
|  |  | (*b*) Provide in the abstract an informative and balanced summary of what was done and what was found | Abstract, pages 2 – 3 |
| Introduction | | |  |
| Background/rationale | 2 | Explain the scientific background and rationale for the investigation being reported | Abstract, page 2, paragraph 1  Background, pages 5-6 |
| Objectives | 3 | State specific objectives, including any prespecified hypotheses | Abstract, page 2, paragraph 1  Background, pages 5-6, paragraph 3 |
| Methods | | |  |
| Study design | 4 | Present key elements of study design early in the paper | Abstract, page 2, paragraph 2  Methods, pages 7-8, paragraphs 1 – 4 |
| Setting | 5 | Describe the setting, locations, and relevant dates, including periods of recruitment, exposure, follow-up, and data collection | Methods, page 7 – 8, paragraphs 1-3 |
| Participants | 6 | (*a*) Give the eligibility criteria, and the sources and methods of selection of participants. Describe methods of follow-up | Methods, pages 7 – 8, paragraph 1 – 4 |
|  |  | (*b*) For matched studies, give matching criteria and number of exposed and unexposed | NA |
| Variables | 7 | Clearly define all outcomes, exposures, predictors, potential confounders, and effect modifiers. Give diagnostic criteria, if applicable | Abstract, page 2, paragraph 2  Methods, page 8 – 10, paragraphs 5 - 8.  Results, paragraph 3, subheading “Adjustment for potential confounders”, page 15-16 |
| Data sources/ measurement | 8* | For each variable of interest, give sources of data and details of methods of assessment (measurement). Describe comparability of assessment methods if there is more than one group | Methods, pages 7 – 8, paragraphs 1, 2, 5 – 6 |
| Bias | 9 | Describe any efforts to address potential sources of bias | Methods, page 7 – 8, 10, paragraphs 2 – 3, 8  Discussion, “strengths and limitations”, pages 23-25 |
| Study size | 10 | Explain how the study size was arrived at | Methods, paragraphs 1 – 4, pages 7-8;  Appendix 1, Figure S1, page 35 |
| Quantitative variables | 11 | Explain how quantitative variables were handled in the analyses. If applicable, describe which groupings were chosen and why | Methods, subheading “Calculating fetal growth velocity” pages 8-9 paragraphs 5-6; and subheading “Statistical analysis”, pages 9 – 10, paragraphs 8 – 10 |
| Statistical methods | 12 | (*a*) Describe all statistical methods, including those used to control for confounding | Methods, subheading “Statistical analysis”, pages 9 – 10, paragraphs 8 – 11 |
|  |  | (*b*) Describe any methods used to examine subgroups and interactions | Methods, subheading “Statistical analysis”, pages 9 – 10, paragraphs 8 – 10 |
|  |  | (*c*) Explain how missing data were addressed | Methods, subheading “Study population and design”, page 7, paragraph 4  Appendix 1, Figure S1, page 35 |
|  |  | (*d*) If applicable, explain how loss to follow-up was addressed | NA |
|  |  | (*e*) Describe any sensitivity analyses | Methods, subheading “Statistical analysis”, page 10, paragraph 9 |
| Results | | |  |
| Participants | 13* | (a) Report numbers of individuals at each stage of study—eg numbers potentially eligible, examined for eligibility, confirmed eligible, included in the study, completing follow-up, and analysed | Results, subheading “Cohort characteristics”, page 12, paragraph 1  Results, Table 1, page 13 – 14  Appendix 1, Figure S1, page 35 |
|  |  | (b) Give reasons for non-participation at each stage | Appendix 1, Figure S1, page 35 |
|  |  | (c) Consider use of a flow diagram | Appendix 1, Figure S1, page 35 |
| Descriptive data | 14* | (a) Give characteristics of study participants (eg demographic, clinical, social) and information on exposures and potential confounders | Results, subheading “Cohort characteristics”, page 12, paragraph 1  Results, Table 1, page 13 – 14 |
|  |  | (b) Indicate number of participants with missing data for each variable of interest | Appendix 1, Figure S1, page 35 |
|  |  | (c) Summarise follow-up time (eg, average and total amount) | NA |
| Outcome data | 15* | Report numbers of outcome events or summary measures over time | Results, subheading “Cohort characteristics”, page 12, paragraph 1  Results, Table 1, page 13 – 14 |
| Main results | 16 | (*a*) Give unadjusted estimates and, if applicable, confounder-adjusted estimates and their precision (eg, 95% confidence interval). Make clear which confounders were adjusted for and why they were included | Results, pages 14 – 16, paragraphs 2 – 5  Results, Table 2, page 15 |
|  |  | (*b*) Report category boundaries when continuous variables were categorized | Results, pages 17-19, paragraphs 6, 7, 9, 10  Results, Table 3, page 19 |
|  |  | (*c*) If relevant, consider translating estimates of relative risk into absolute risk for a meaningful time period | NA |
| Other analyses | 17 | Report other analyses done—eg analyses of subgroups and interactions, and sensitivity analyses | Results, pages 16 – 19, paragraphs 4 – 10  Results, Table 3, page 19 |
| Discussion | | |  |
| Key results | 18 | Summarise key results with reference to study objectives | Discussion, subheading “Main findings”, pages 20 – 21 |
| Limitations | 19 | Discuss limitations of the study, taking into account sources of potential bias or imprecision. Discuss both direction and magnitude of any potential bias | Discussion, subheading “Strengths and limitations”, pages 23 – 25 |
| Interpretation | 20 | Give a cautious overall interpretation of results considering objectives, limitations, multiplicity of analyses, results from similar studies, and other relevant evidence | Discussion, subheading “Interpretation of results and comparison with other studies”, pages 21 – 23  Discussion subheading “Clinical implications”  Pages 25 – 26  Conclusions, Page 27 |
| Generalisability | 21 | Discuss the generalisability (external validity) of the study results | Discussion subheading “Strengths and limitations”, paragraph 3, page 24  Discussion, subheading “Clinical implications”, pages 25 – 26  Conclusions, page 27 |
| Other information | | |  |
| Funding | 22 | Give the source of funding and the role of the funders for the present study and, if applicable, for the original study on which the present article is based | Declarations, subheading “Funding”, page 29 |

*Give information separately for exposed and unexposed groups.

**Note:** An Explanation and Elaboration article discusses each checklist item and gives methodological background and published examples of transparent reporting. The STROBE checklist is best used in conjunction with this article (freely available on the Web sites of PLoS Medicine at http://www.plosmedicine.org/, Annals of Internal Medicine at http://www.annals.org/, and Epidemiology at http://www.epidem.com/). Information on the STROBE Initiative is available at http://www.strobe-statement.org.
